# Academic Stressors and Sociodemographic Vulnerabilities Among Medical Students at the University of Ottawa

**DOI:** 10.12688/mep.21466.1

**Published:** 2026-02-27

**Authors:** Sierra A. Land, Hailey C. Land, Jordyn N. Linders, Aly Julien, Kay-Anne Haykal

**Affiliations:** 1 University of Ottawa Faculty of Medicine, Ottawa, Ontario, Canada; 2 Institut du Savoir Montfort, Ottawa, Ontario, K1K 0T2, Canada

**Keywords:** medical students, stressors, sociodemographic factors, undergraduate medical education

## Abstract

**Introduction:**

Medical students experience high levels of stress that impact their health and academic performance. Yet, to our knowledge, no prior Canadian study has explored medical training-related stressors, using a validated tool such as the Medical Student Stress Questionnaire, leaving a gap in the national literature. The objective of this study was to determine the major academic stressors and their relationship with sociodemographic factors among medical students at the University of Ottawa.

**Methods:**

This cross-sectional study was conducted using a SurveyMonkey questionnaire from May to August 2024 with the 40 items of the Medical Student Stress Questionnaire. Variables were age, year of study, gender, LGBTQ2S+, ethnicity, rural/urban, living situation, financial situation, disability, and dependents. Descriptive statistics, t-tests, ANOVAs, and multiple regression analyses were conducted using SPSS v30.0. The study was approved by the University of Ottawa Research Ethics Board.

**Results:**

70 medical students participated, but 60 complete responses were analyzed (85.7%). The mean total Medical Student Stress Questionnaire score was 2.63 ± 0.79, i.e. moderate stress. Academic-related stressors were the highest, particularly high workload, pressure to perform, and frequency of tests and exams. Preclinical students reported higher stressed across all domains. Male students had higher academic-related stressors (p = 0.006), and students with disabilities had higher stress from group work (p = 0.034). Living with family predicted higher teaching and learning stress (p = 0.005).

**Discussion:**

Stress is common in medical trainees, with domain-specific vulnerabilities indicating the need for universal and targeted interventions to support equity and learner well-being in undergraduate medical education.

## Introduction

Canadian medical students encounter high levels of stress throughout their training. ^1^ A recent study at the University of Calgary surveying 101 medical students early in the COVID-19 pandemic reported that 81% identified their studies as a major stressor, 21% reported a current mental health condition, 35% experienced financial stress, and 75% met the exhaustion threshold on the *Oldenburg Burnout Inventory.* ^1^ Consistent with these findings, a multicentre study reported that 37% of Canadian medical students met criteria for burnout. ^2^ A recent study conducted in Ontario found that medical students demonstrated significant levels of burnout on the *Copenhagen Burnout Inventory*, with fourth-year students in particular reporting very high levels of stress and anxiety. ^3^

International studies demonstrate that elevated stress among medical trainees is associated with burnout, reduced empathy, professional misconduct, depression, and suicidal ideation. ^4– 6^ Several investigations have further explored sociodemographic influences on student well-being. ^6– 8^ For example, gender, financial status, and nationality predicted health outcomes among medical students in Germany and Hungary ^8^; in Pakistan, students from low-income families reported higher burnout ^6^; and in Serbia, older age and self-financed education were associated with increased burnout. ^7^

In Canada, medical students’ wellness has become a national priority. The Association of Faculties of Medicine of Canada has created a working group on learner well-being to promote best practices in medical schools. ^9^ These initiatives show that importance is given to the mental health of learners as an integral part of the sustainability of the medical workforce and patient safety. Indeed, student distress has been associated with decreased clinical performance, medical errors, and reduced patient satisfaction. ^10^

Despite this growing attention, Canadian data remain limited, particularly regarding how stress manifests across sociodemographic groups. Addressing this gap is important, as wellness and equity are increasingly emphasized as core competencies in undergraduate medical education. ^9^ Moreover, much of the existing literature relies on general psychological distress measures, rather than identifying specific training-related stressors that can inform targeted curricular and institutional interventions. ^5^ To our knowledge, no study conducted in Canada has measured the stressors experienced by medical students using a validated tool, such as the Medical Student Stress Questionnaire (MSSQ). Research conducted in Canada has focused primarily on overall psychological distress and burnout among medical students, without identifying which aspects of the curriculum are the most stressful. ^3, 11, 12^ A study highlights the influence of factors such as gender, socioeconomic status, and cultural identity on the mental health of medical students, but does not address the stressors associated with their training. ^13^ The lack of data in Canada combining a validated instrument for measuring academic stressors and the sociodemographic profile of students is a significant gap in the current literature.

The University of Ottawa, as Canada’s only university with a bilingual medical school, offers a uniquely diverse learning environment, making it an ideal setting to explore how stress experiences vary across linguistic, cultural, and demographic dimensions. ^14, 15^ Accordingly, this study aims to identify, quantify, and compare training-related stressors and to examine associations with sociodemographic variables among Undergraduate Medical Education (UGME) students at the Faculty of Medicine of University of Ottawa.

## Methods

### Study design

This cross-sectional observational study was conducted at the Faculty of Medicine of University of Ottawa between May 1 and August 30, 2024. The study followed the STROBE (Strengthening the Reporting of Observational Studies in Epidemiology) checklist for cross-sectional studies. ^16^

### Ethics

Ethical approval was obtained from the University of Ottawa Research Ethics Board (REB #H-02-24-10066). After obtaining ethical approval, every participant gave their digital informed consent. The first page of the online survey included the consent statement. Students who chose “agree” kept going with the survey, but those who chose “disagree” left the form and were thanked with no further action required. The consent letter explained to the participants what the study was about, what the risks were, what their rights were, and how to contact the main investigator. The document established out the criteria for who could and couldn’t take part, making it clear that anyone could withdraw at any time.

### Transparency and open science

In accordance with open science practices and to promote transparency, a preliminary version of this study was made available as a preprint. ^17^

### Participants and recruitment

An anonymous bilingual online survey was administered via SurveyMonkey to Undergraduate Medical Education MD students (graduating cohorts 2024–2027) and MD/PhD students (pre-PhD or post-PhD phase) at the Faculty of Medicine of University of Ottawa. Eligibility criteria included current enrollment in either the anglophone or francophone streams. Students on a formal leave of absence were excluded. The survey link was distributed through institutional email and private class Facebook groups, with support from student representatives. Participation in this study was entirely voluntary and incompletely filled-out questionnaires were excluded from statistical analysis. The survey required approximately 10-12 minutes to complete, and no identifying data or IP addresses were collected to ensure anonymity.

### Variables

Sociodemographic variables were selected based on prior literature linking them to stress and burnout among medical learners. ^6, 18^ Variables included age, year of study, sex assigned at birth, LGBTQ2S+ identity, ethnicity, rural versus urban upbringing, current living situation, home distance from campus, household income, financial status, non-academic work hours, disability status, and number of dependents.

### Data sources and measurement

The survey incorporated the 40-item of the MSSQ, a validated instrument measuring stressors across six domains: academic-related stressors (ARS), intrapersonal and interpersonal-related stressors (IRS), teaching- and learning-related stressors (TLRS), social-related stressors (SRS), drive- and desire-related stressors (DRS), and group activities-related stressors (GARS). Each item was rated on a 5-point Likert scale from 0 (causing no stress) to 4 (causing severe stress).

The MSSQ has demonstrated strong validity across international contexts. For example, in Sri Lanka the adapted MSSQ showed a Cronbach’s alpha of 0.95 and test-retest correlation of 0.918 (p < 0.001) in a 603-student sample. ^19^ In Turkey, confirmatory factor analysis among 412 students yielded RMSEA = 0.060, CFI = 0.899, and total-scale α = 0.94. ^20^ In our study, Cronbach’s alpha was 0.951, indicating excellent internal consistency across the six domains.

### Study size

As this was an exploratory cross-sectional study using a convenience sample, the sample size was based on the number of students who chose to participate during the recruitment period rather than on a formal sample size calculation. In descriptive research using non-probability sampling, sample size planning is commonly not based on statistical formulas but driven by feasibility and voluntary participation within the study timeframe. ^21^

### Statistical analysis

Using Microsoft Excel 2025, the data were cleaned, coded, and exported to SPSS version 30.0 (IBM Corp.) for analysis. Descriptive statistics summarized participant demographics and MSSQ domain scores. The average scores per domain were classified as mild (0.00-1.00), moderate (1.01-2.00), high (2.01-3.00), or severe (3.01-4.00). Comparisons between groups were made using independent samples t-tests and one-way ANOVA. Pearson correlations and multiple linear regressions were used to examine associations between stress scores and sociodemographic predictors. Interaction terms were examined to explore differences between subgroups. Sensitivity analyses were performed by comparing models with and without grouped categories with low cell counts (n < 5).

Missing data were handled using pairwise deletion to preserve statistical power. MSSQ scores were computed only for participants who completed at least 95% of items, consistent with prior validation studies. To maintain analytical stability, several demographic variables were collapsed into fewer categories: age (<25 vs. ≥25 years), year of study (Pre-Clerkship: Years 1–2 vs. Clerkship: Years 3–4), non-academic work hours (0–9 vs. 10–39 hours/week), and household income (<$150,000 vs. ≥$150,000).

Sex assigned at birth and LGBTQ2S+ identity were treated as binary variables, and disability status was yes, no, or prefer not to say. Sources of funding were counted from 1 to 6 based on the number of options chosen (e.g., loans, family, scholarships, savings, etc.). Community upbringing was categorized as urban, suburban, or rural/remote based on self-reported high school location. Home distance from campus was categorized as <50 km, ≥50 km, or out of province. Current living situation was classified as independent, student housing, with family/guardian/significant other, or other; all categories were retained for descriptive reporting. Ethnic identity was collapsed into three mutually exclusive groups: “White,” “Other ethnic groups” (e.g., Asian, Middle Eastern, Indigenous), and “No response.” For multi-select responses, the least-represented non-White identity was retained to enhance visibility of equity-deserving groups.

### Bias

To minimize bias, selection bias was reduced by including all eligible students and ensuring voluntary participation, measurement bias was minimized by using the validated and standardized MSSQ, and social desirability bias was limited by collecting responses anonymously, without any identifying information.

## Results

### Sample demographics

A total of 70 students participated, with 60 complete responses analyzed (85.7%). About half (45.0%) were under 25 and 51.7% were in pre-clerkship. A majority identified as male (83.3%), and 16.7% identified as LGBTQ2S+. Two-thirds (65.0%) identified as White and 31.7% as another ethnic group. Most students reported suburban (56.7%) or urban (28.3%) backgrounds. Living situations varied, with 40.0% living with peers, 33.3% independently, and 23.3% with family. Household income during high school was below $150,000 for 60.0% of respondents. Students reported multiple financial support sources, most commonly three. Half worked 0–9 hours per week, and half 10–39 hours. Nine students (15.0%) reported a disability, and four (6.7%) had dependents ( Table 1).

**Table 1.** Sociodemographic characteristics of respondents (N = 60).

Variable	Category	n (%)
Age	< 25 years	27 (45.0)
	≥ 25 years	33 (55.0)
Program stage	Pre-clerkship	31 (51.7)
	Clerkship	29 (48.3)
Gender	Male	50 (83.3)
	Female	10 (16.7)
Sexual orientation	LGBTQ2S+	10 (16.7)
	Not LGBTQ2S+	49 (81.7)
	Prefer not to say	1 (1.7)
Race/Ethnicity	White	39 (65.0)
	Other ethnic group *	19 (31.7)
	Prefer not to say	2 (3.3)
High school location	Suburban	34 (56.7)
	Urban	17 (28.3)
	Rural/remote	9 (15.0)
Current living situation	With peers	24 (40.0)
	Independently	20 (33.3)
	With family/partner	14 (23.3)
	Other	2 (3.3)
Home distance from campus	< 50 km	28 (46.7)
	≥ 50 km	25 (41.7)
	Out of province	7 (11.7)
Household income (high school)	< $150,000	36 (60.0)
	≥ $150,000	24 (40.0)
Financial support sources	One	8 (13.3)
	Two	14 (23.3)
	Three	21 (35.0)
	Four	10 (16.7)
	Five	5 (8.3)
	Six	2 (3.3)
Non-academic work hours	0–9 hours/week	30 (50.0)
	10–39 hours/week	30 (50.0)
Disability	Yes	9 (15.0)
	No	49 (81.7)
	Prefer not to say	2 (3.3)
Dependents	Yes	4 (6.7)
	No	56 (93.3)

* Includes East Asian, South Asian, Southeast Asian, Middle Eastern, or Indigenous (First Nations, Inuit, Métis) identity.

### Overview of peak stressors across the cohort

The most prominent stressors experienced by medical students across the cohort were primarily academic-related stressors ( Table 2). The highest mean stressor was “Large amount of content to be learned” (mean = 2.62, SD = 0.99), closely followed by “Need to do well (self-expectation)” (mean = 2.60, SD = 1.18). Other commonly supported academic stressors were “Workload” (mean = 2.43, SD = 0.91), “Exams and tests” (mean = 2.43, SD = 0.96), and “Not enough time to go over what we have learned” (mean = 2.28, SD = 1.15). Besides cognitive stressors, students also mentioned time and skill-related stressors, namely “Lack of time for family and friends” (mean = 2.18, SD = 1.07) and “Not enough medical skill practice” (mean = 2.18, SD = 1.16) as the top stressors. Emotional and interpersonal stressors also appeared, with “Uncertainty of what is expected of me” (mean = 1.93, SD = 1.22), “Feeling of incompetence” (mean = 1.95, SD = 1.31), and “Grading” (mean = 1.78, SD = 1.17) rounding out the top ten.

**Table 2.** Top ten most prominent stressors reported by students with highest mean scores by sociodemographic factor (n > 5).

Stressor	Mean ± SD	Highest-scoring sociodemographic variable	Group (n > 5)	Group mean	p-value
Large amount of content to be learned	2.62 ± 0.99	Living situation	Living with family (n = 14)	3.07	.282
Need to do well (self-expectation)	2.60 ± 1.18	Ethnicity	White (n = 39)	2.72	.520
Workload	2.43 ± 0.91	Community upbringing	Rural/remote (n = 9)	2.78	.472
Exams and tests	2.43 ± 0.96	Age	< 25 years (n = 27)	2.70	.050
Lack of time for family/friends	2.18 ± 1.07	Living situation	Living with family (n = 14)	2.57	.285
Not enough medical skill practice	2.18 ± 1.16	Living situation	Living with family (n = 14)	2.64	.273
Lack of time to review	2.28 ± 1.15	Financial support sources	Two sources (n = 14)	2.79	.246
Uncertainty of expectations	1.93 ± 1.22	Disability status	Has disability (n = 9)	2.44	.111
Feeling of incompetence	1.95 ± 1.31	Disability status	Has disability (n = 9)	2.67	.068
Grading	1.78 ± 1.17	Disability status	Has disability (n = 9)	2.44	.180

P-values based on independent samples t-tests or ANOVA; α = .05. Only sociodemographic groups with n > 5 are reported.

The stressor “Large amount of content to be learned” was highest among students living with family (mean = 3.07, n = 14), although this difference was not statistically significant (p = 0.282). The highest mean score for “Need to do well” was observed among students identifying as White (mean = 2.72, n = 39; p = 0.520), and for “Workload” among students with a rural/remote upbringing (mean = 2.78, n = 9; p = 0.472); neither reached statistical significance. Students younger than 25 reported the highest mean stress scores for “Exams and tests” (mean = 2.70, n = 27), which approached statistical significance (p = 0.050). The stressors “Lack of time for family and friends” (mean = 2.57, n = 14; p = 0.285) and “Not enough medical-skill practice” (mean = 2.64, n = 14; p = 0.273) were both highest among students living with family, though neither was statistically significant. Students with two financial support sources reported the highest mean for “Lack of time to review what we have learned” (mean = 2.79, n = 14; p = 0.246), again not significant. The highest means for “Uncertainty of what is expected of me” (mean = 2.44, n = 9; p = 0.111), “Feeling of incompetence” (mean = 2.67, n = 9; p = 0.068), and “Grading” (mean = 2.44, n = 9; p = 0.180) were all observed among students identifying as having a disability, but none reached statistical significance.

### Variation in stressors across sociodemographic groups

When analyzing sociodemographic variables further, several trends arose across domains and stressor subcategories emerged, though few reached statistical significance ( Table 3). By age group, students under 25 reported higher stress across most stressor subcategories, with “Exams and tests” approaching significance (mean = 2.70 versus 2.21, p = 0.05); all other comparisons were not statistically significant (p ≥ 0.10). By academic year, pre-clerkship students reported higher stress than clerkship students in the following stressor subcategories: “Need to do well” (p = 0.03), “Not enough medical skill practice” (p < 0.01), “Lack of time to review what we have learned” (p = 0.04), “Uncertainty of what is expected of me” (p < 0.01), and “Grading” (p = 0.03). In sex, male students had higher ARS than females (mean = 2.12, p = 0.006), higher stress for “Exams and tests” (p = 0.002) and “Lack of time to review what we have learned” (p = 0.002). With respect to LGBTQ2S+ identity, there were no significant differences (p > 0.06).

**Table 3.** Mean domain scores on the MSSQ and associated sociodemographic groups with highest reported stress (n > 5).

MSSQ domain	Mean ± SD	Highest-scoring sociodemographic group (n > 5)	Group mean	p-value
Academic-Related Stressors (ARS)	1.99 ± 0.82	Male (n = 50)	2.12	.006
Intrapersonal & Interpersonal Stressors (IRS)	0.57 ± 0.48	White students (n = 39)	0.62	.236
Teaching & Learning-Related Stressors (TLRS)	1.39 ± 1.02	Living with family (n = 14)	1.80	.005
Social-Related Stressors (SRS)	1.25 ± 0.67	White students (n = 39)	1.31	.174
Drive & Desire-Related Stressors (DRS)	0.68 ± 0.73	White students (n = 39)	0.74	.485
Group Activities-Related Stressors (GARS)	1.28 ± 0.92	Students with disability (n = 9)	1.94	.034

P-values from independent-samples t-tests or ANOVA. α = .05. Only groups with n > 5 are displayed.

For ethnicity, White students had the highest mean scores across IRS, SRS, and DRS domains, but ethnicity was ultimately not significantly associated with stress in any category (p > 0.17). By high school community type, rural/remote and suburban students reported higher stress in many stressor subcategories, such as “Need to do well,” yet these differences were not statistically significant (p > 0.14). Regarding living arrangement, students staying with family had higher TLRS (p = 0.005). For home distance from campus, stressor subcategory scores were not significantly different (p ≥ 0.19). Household income and number of financial support sources were not statistically significant (p > 0.20). For non-academic hours, students working 10-39 hours per week had slightly higher stress in most areas, but not significantly (p > 0.20). Students with disabilities reported significantly higher GARS (p = 0.034), with trend-level elevation in “Feeling of incompetence” (p = 0.07), with all other comparisons not statistically significant.

## Discussion

This study investigated the prevalence and patterns of academic stressors in medical students at a Canadian bilingual university and associations with sociodemographic factors. In line with previous studies, we observed that stress was common and mainly caused by ARS, such as the amount of content, performance, workload, and frequent exams. These findings are consistent with other research demonstrating that academic overload remains a significant source of distress in undergraduate medical education and contributes significantly to emotional exhaustion, decreased empathy, and lower academic performance. ^1, 22^ The prevalence of ARS in almost all demographic subgroups suggests that systemic demands of the curriculum continue to greatly influence student well-being, increasing the need for institutional interventions aimed at addressing structural factors contributing to stress, rather than placing the responsibility solely on individual coping strategies. ^23– 25^

Universally targeted academic supports, such as early skills-building, time-management strategies, mentorship, and transparent assessment expectations, may therefore benefit the entire learner population. Initiatives such as uOttawa’s Student Academic Success Service and Faculty of Medicine academic remediation programs already provide relevant infrastructure. ^23, 24^ Nationally, Canadian medical schools have adopted wellness frameworks aligned with the Canadian Federation of Medical Students recommendations ^25^ and examples such as the University of Calgary’s WISHES program, ^26^ which supports academic resilience, emotional well-being, and social connection. But disparities in uptake and integration suggest that wellness systems need to go further upstream, to curricular and policy-level change.

Beyond systemic challenges, subgroup gaps highlight the need for targeted supports. Pre-clerkship students, in our study, had greater stress in areas such as uncertainty of expectations, lack of medical skills practice, and academic pressure, which is consistent with the steep cognitive and emotional learning curve of medical school. ^1, 27, 28^ Stress levels are also high among younger students, particularly with regard to exams, which is consistent with the findings of other studies suggesting that maturity and university experience can improve coping mechanisms over time. ^1, 27, 28^

In our study, male students reported higher levels of ARS than female students, especially about exam-related stress and limited time for revision. This contradicts existing literature, according to which female trainees report higher levels of stress and burnout. ^18^ To explain this, we can point to the underuse of support resources among male students, differences in help-seeking behavior, and gender imbalance in our sample (50 male respondents, 10 female respondents).

Living context also emerged as a meaningful determinant: students residing with family experienced greater teaching- and learning-related stress (TLRS) and greater concern about grading. This is consistent with studies indicating that family pressure, ^29^ lack of study privacy, and intergenerational expectations can exacerbate academic stress. ^30^ In contrast, students with disabilities reported higher group-activities-related stress, with trends toward higher incompetence. This parallels research indicating that disabled students experience greater psychological distress, administrative burden in obtaining accommodations, and fear of judgement, ^31, 32^ highlighting the need for proactive accessibility policies and stigma-free support routes in medical education.

However, other frequently reported predictors of stress such as ethnicity, LGBTQ2S+ status, financial factors, and geographic origin were not significantly related to stress in this study. This differs from international work linking financial hardship and minority identity to greater burnout and psychological distress. ^6, 8, 27, 33, 34^ These differences may be due to contextual equity policies at the University of Ottawa, the predominance of academic variables, or insufficient statistical power to detect small or intersectional effects in our study. Some stressors, like subtle discrimination, microaggressions, and language stress in bilingual contexts, might not be fully captured by the MSSQ and would benefit from qualitative investigation. ^35^ As such, the absence of statistical significance should not be interpreted as absence of effect; instead, it highlights the need for more granular and mixed-methods approaches to equity-focused medical education research in Canada.

### Limitations

This study offers novel perspectives on stress and sociodemographic factors in medical students at a bilingual Canadian university. But there are limitations. Firstly, the sample was taken from one university, which limits generalizability to other medical training programs in Canada. The small sample size may reduce statistical power and increase non-response bias, and the overrepresentation of males may obscure patterns among students of different genders. Second, stress levels may have also varied depending on the time of the survey, which was during exam periods and summer. Additionally, this is a cross-sectional, self-report study, so causality cannot be established, and responses may be subject to recall and social desirability biases. Finally, although the MSSQ is validated, but it does not consider cultural or contextual stressors (e.g., microaggressions, debt-related stress, language requirements), which may be particularly relevant in bilingual and diverse learning environments.

### Implications and future directions

The results of our study showed that some medical students are more vulnerable to stress, indicating the importance of having support programs in medical schools, such as mental health clinics, tailored to their needs. Medical schools in Canada should increase mental health resources and offer personalized support to students who are unable to effectively manage academic-related stressors. Further research could focus on a larger sample, bringing together several medical schools in Canada, examining the evolution of stress during medical training, and analyzing how certain therapies can alleviate the stress associated with medical training.

## Conclusion

To improve well-being, academic success, and equity in medical education, we need to understand stress and its sociodemographic correlates. Academic stressors remained the most common stressor in our study. Although statistically significant associations between sociodemographic variables were few, our results highlight specific vulnerabilities in certain areas among younger students, male trainees, students with disabilities, and those living with family. This study complements the Canadian literature on medical education by providing one of the first domain-specific analyses of academic stressors using a validated tool in a bilingual training environment. These results are useful for those who support medical students daily, such as undergraduate medical education administrators, student affairs, and curriculum committees. Longitudinal studies tracking students through medical school could reveal how stress changes over time and how students experience it in different learning contexts.

## Data Availability

The datasets used and analyzed in this study are not publicly available for ethical and confidentiality reasons concerning the participants. They are stored on the University of Ottawa’s institutional shared drive. Anonymized or aggregated data may be disclosed upon reasonable request to Dr. Kay-Anne Haykal ( khaykal@uottawa.ca), subject to obtaining ethical approval and complying with confidentiality rules, and under conditions that guarantee student confidentiality.
