# Supplementary material for: Academic Stressors and Sociodemographic Vulnerabilities Among Medical Students at the University of Ottawa
Source: MedEdPublish (2016). 2026 May 27;16:4. Originally published 2026 Feb 27. [Version 2] doi: 10.12688/mep.21466.2 (PMC13261187; doi:10.12688/mep.21466.2)
Supplement: Peer review history

# Reviewer report: Reviewer response for version 2

**DOI:** 10.21956/mep.23225.r45948

**Published:** 2026-06-11

**Authors:** Alba Madrid Cagigal

**Affiliations:** 1 University of Galway, Galway, County Galway, Ireland

The authors have satisfactorily resolved all of my previous concerns, and I have no further comments.

Have any limitations of the research been acknowledged?

Yes

Is the work clearly and accurately presented and does it cite the current literature?

Yes

If applicable, is the statistical analysis and its interpretation appropriate?

Yes

Are all the source data underlying the results available to ensure full reproducibility?

Partly

Is the study design appropriate and is the work technically sound?

Yes

Are the conclusions drawn adequately supported by the results?

Partly

Are sufficient details of methods and analysis provided to allow replication by others?

Partly

Reviewer Expertise:

Digital Mental Health Interventions for University Students

I confirm that I have read this submission and believe that I have an appropriate level of expertise to confirm that it is of an acceptable scientific standard.

# Reviewer report: Reviewer response for version 2

**DOI:** 10.21956/mep.23225.r45949

**Published:** 2026-06-02

**Authors:** Binata Mukherjee

**Affiliations:** 1 University of South Alabama, Mobile, Alabama, USA

I have no further comments as the authors have addressed my previous questions to my satisfaction.

Have any limitations of the research been acknowledged?

Yes

Is the work clearly and accurately presented and does it cite the current literature?

Yes

If applicable, is the statistical analysis and its interpretation appropriate?

Yes

Are all the source data underlying the results available to ensure full reproducibility?

Yes

Is the study design appropriate and is the work technically sound?

Yes

Are the conclusions drawn adequately supported by the results?

Partly

Are sufficient details of methods and analysis provided to allow replication by others?

Partly

Reviewer Expertise:

Leadership development/coaching/well-being/wellness in academic medicine.

I confirm that I have read this submission and believe that I have an appropriate level of expertise to confirm that it is of an acceptable scientific standard.

# Reviewer report: Reviewer response for version 1

**DOI:** 10.21956/mep.22994.r45713

**Published:** 2026-04-27

**Authors:** Binata Mukherjee

**Affiliations:** 1 University of South Alabama, Mobile, Alabama, USA

Stress, burnout, and well-being for productivity are of significant concern in the medical community in general. These issues start early during student life and percolate throughout the career cycle of physicians. Thank you for studying this relevant topic. The manuscript is well written. My suggestions for revisions are essentially for strengthening the manuscript.

 1. Instrument selection. What other instruments are available for measuring students’ stress that might have been used? Please explain why you chose this instrument over others.

 2. Please include the STROBE checklist with reference to your article in the supplement.

 3. Statistical analysis. You mentioned Pearson correlations and multiple linear regressions were used to examine associations between stress scores and sociodemographic predictors…..Which sociodemographic predictors did you choose?

 4. What was the total number of recipients? What was the response rate from that total pool of recipients? How does the respondent breakdown compare with the total student body of the school in terms of age, gender, sexual orientation, and ethnicity? Considering that more male students responded to the survey, it would be interesting to have this background information.

 5. Since the results are summarized in Table 1, I would recommend removing the line-by-line narrative of the results in the text. The reader can see the results in the table.

 6. I would have liked to see a table for the stressors breakdown by pre-clerkship/clerkship and by age since you noted interesting distinctions in the performance amongst these groups.

 7. Table 3. It would be interesting to include the domain specific results for all the sociodemographic groups; the readers are kept in the dark about the performance of the overall student population; I didn’t find that in your article.

 8. Implications and future directions. Considering that ARS and TLRS show significant p values, you may want to make recommendations specifically addressing these two. I would encourage you to be more specific in your recommendations.

 To conclude, this is a well written manuscript that should help medical educators and leaders.

Have any limitations of the research been acknowledged?

Yes

Is the work clearly and accurately presented and does it cite the current literature?

Yes

If applicable, is the statistical analysis and its interpretation appropriate?

Yes

Are all the source data underlying the results available to ensure full reproducibility?

Yes

Is the study design appropriate and is the work technically sound?

Yes

Are the conclusions drawn adequately supported by the results?

Partly

Are sufficient details of methods and analysis provided to allow replication by others?

Partly

Reviewer Expertise:

Leadership development/coaching/well-being/wellness in academic medicine.

I confirm that I have read this submission and believe that I have an appropriate level of expertise to confirm that it is of an acceptable scientific standard.

# Reviewer report: Reviewer response for version 1

**DOI:** 10.21956/mep.22994.r45484

**Published:** 2026-03-13

**Authors:** Alba Madrid Cagigal

**Affiliations:** 1 University of Galway, Galway, County Galway, Ireland

Thank you for the opportunity to review this interesting and relevant manuscript addressing an important issue in medical education and contributing to the limited Canadian literature on medical student stressors using a validated instrument. The study is clearly written and the research objective is well defined. I have outlined several areas where clarification or additional detail may help strengthen the manuscript.

**1. Introduction**

 The introduction provides a clear overview of the issue of stress among medical students and appropriately situates the study within the existing literature. The research gap (limited Canadian studies using the Medical Student Stress Questionnaire) is well articulated and provides a strong rationale for the study.

 - The introduction could be strengthened by more explicitly explaining how identifying domain-specific stressors can inform interventions in medical education.

 - The authors may wish to provide a brief explanation of why the MSSQ was selected for this study. While the instrument is described later in the Methods section, including a short justification in the introduction would further strengthen the rationale for the study design.

**2. Methods**

 Overall, the methods are clearly described. However, several aspects of the recruitment strategy and methodological transparency would benefit from further clarification.

 - The recruitment strategy could be described in greater detail. Because the survey link was distributed through institutional email and private class Facebook groups, it is not entirely clear how participant eligibility was verified.

 - The manuscript could clarify whether participants received any form of incentive or compensation for completing the survey.

 - Although the authors note that the study used a convenience sample, methodological transparency could be strengthened by discussing the implications of the relatively small sample size for statistical power.

**3. Results**

 The results are presented clearly and provide a useful overview of the main stressors experienced by participants. However, the demographic distribution of the sample appears somewhat skewed, particularly with respect to sex, with 83% of respondents identifying as male.

 - The authors may wish to discuss potential reasons for this imbalance and consider how sampling bias or differential participation may have influenced the findings.

**4. Discussion**

 Overall, the discussion is well written and effectively situates the findings within the broader literature on medical student stress. 

 - As outlined above, the manuscript could benefit from expanding the discussion of how the overrepresentation of male respondents may have influenced the results.

 - The statement suggesting that this finding may be explained by the underuse of support resources among male students would benefit from supporting references.

 - The implications section could be made more specific. While the manuscript suggests increasing mental health resources and support programs, it may be helpful to provide more concrete examples of potential interventions

Have any limitations of the research been acknowledged?

Yes

Is the work clearly and accurately presented and does it cite the current literature?

Yes

If applicable, is the statistical analysis and its interpretation appropriate?

Yes

Are all the source data underlying the results available to ensure full reproducibility?

Partly

Is the study design appropriate and is the work technically sound?

Yes

Are the conclusions drawn adequately supported by the results?

Partly

Are sufficient details of methods and analysis provided to allow replication by others?

Partly

Reviewer Expertise:

Digital Mental Health Interventions for University Students

I confirm that I have read this submission and believe that I have an appropriate level of expertise to confirm that it is of an acceptable scientific standard, however I have significant reservations, as outlined above.
